# Editorial: Transcription and its regulation in bacteria

**DOI:** 10.3389/fmicb.2023.1200443

**Published:** 2023-05-12

**Authors:** Dong Wang, Dmytro Kompaniiets, Yangbo Hu, Bin Liu

**Affiliations:** ^1^Section of Transcription and Gene Regulation, The Hormel Institute, University of Minnesota, Austin, MN, United States; ^2^State Key Laboratory of Virology, Wuhan Institute of Virology, Center for Biosafety Mega-Science, Chinese Academy of Sciences, Wuhan, China

**Keywords:** transcription, RNA polymerase, transcription factors, transcription regulation, gene expression, DNA promoter

Transcription is the fundamental process in all cells, in which the genetic information is copied from a DNA template into Messager RNA. In bacteria, the evolutionarily conserved multi-subunit DNA-dependent RNA polymerase (RNAP) is the essential enzyme that is responsible for transcription. As the first and central step of gene expression, transcription is regulated by a variety of transcriptional factors. In addition, bacterial RNAP is not only an ideal research model for all cellular RNAPs, but also an important target for antibacterial drug development. Thus, understanding bacterial transcription and how it is regulated by various transcriptional factors is significant and greatly benefits public health.

Transcriptional factors that integrate cellular and environmental signals to control cell biology are well known in bacteria. By targeting RNAP or/and DNA promoters, they can switch on or off the expression of genes, enabling bacteria to adapt to and survive under stress conditions. These factors are often referred to as “global regulators” because they can control the expression of many genes. Some of these regulators can also integrate multiple signals to fine-tune gene expression, allowing the bacteria to respond to complex and dynamic environmental conditions. The main objective of this Research Topic was to uncover the role of transcriptional factors in bacteria and their response to cellular changes, with the aim of enhancing our understanding of the pathogenicity mechanisms in various bacterial species. Recently, four articles were published on this topic, providing complementary insights into the roles of global transcriptional factors in bacterial adaptation and virulence.

Pellegrini et al. studied the role of transcriptional factor CodY in controlling and coordinating metabolism and virulence in Group B Streptococcus (GBS). They confirmed that CodY is required for GBS infection in neonatal and adult animal models but not essential for the growth of GBS in complex or chemically defined liquid medium. Deletion of *codY* gene decreased *in vivo* lethality, which was related to an impaired ability of the mutant to persist in the blood, spread to distant organs, and cross the blood-brain barrier. In addition, CodY contributes to GBS adhesion to epithelial cells and controls GBS biofilms formation. In light of transcriptomic analysis, CodY was found to regulate ~13% of GBS's genome, primarily functioning as a suppressor of genes that are associated with amino acid transportation and metabolism, as well as those encoding surface-anchored proteins. Interestingly, in contrast to the decreased virulence and ability to cross blood-brain barrier in *codY* deleted strain, level of Srr2 is significantly increased, suggesting involvement of additional regulatory factor. The activity of CodY was also shown to be reliant upon the presence of branched-chain amino acids, which act as the universal cofactors for this regulator. In this work, the authors provided a detailed study on the roles of CodY controlling GBS virulence.

Reprogramming of virulence gene expression is crucial for bacterial survival and adaptation to different host environments. Pettersen et al. investigated the global transcriptional responses of *Streptococcus pneumoniae* (pneumococcus) to human blood components and cerebrospinal fluid (CSF) acquired from discarded and anonymized patient samples. The data showed significant changes in the pneumococcal transcriptome during incubation with human blood components and CSF. The cellular components of blood were found to be the main stressors to pneumococcus inside the bloodstream, while plasma components primarily induced a metabolic adaptation response. Notably, changes observed in CSF samples were similar to those observed in plasma samples due to their similarities. Additionally, more than 20 small non-coding RNAs were identified as differentially expressed in reaction to the various conditions, particularly in response to red blood cells. The authors highlighted the involvement of metabolic pathways such as fatty acid and nucleotide biosynthesis, and ncRNA-mediated regulation in pneumococcal virulence.

Bacterial antimicrobial resistance presents a significant threat to global human and animal health, and bacterial metabolism is related to susceptibility and resistance to antibiotics. Mao et al. studied the role of the global transcriptional regulator FNR in metabolism and its contribution to antibiotic resistance in *Edwardsiella tarda*. Deletion of *fnr* gene resulted in increased sensitivity to aminoglycoside antibiotics. This is linked to the most activated alanine, aspartate, and glutamate metabolism and the P cycle, which promoted proton motive force. Notably, exogenous glutamate had a similar effect as *fnr* deletion in promoting sensitivity to aminoglycoside drugs. The study concludes that FNR may regulate glutamate metabolism to contribute to aminoglycoside resistance in *Edwardsiella tarda*.

The work of Zhao et al. focused on the transcriptional regulatory mechanism of MsmR1 in *Paenibacillus polymyxa* SC2 for expanding its potential applications in biological control against specific pathogens in pepper. *MsmR1* deletion was shown to decrease polymyxin synthesis. Further chromatin immunoprecipitation assay with sequencing (ChIP-seq) combined with electromobility shift assays (EMSA) were performed to describe the regulatory network of the global regulator MsmR1, indicating the significant connection between MsmR1 and carbohydrate metabolism pathways. MsmR1 positively regulates polymyxin synthesis by directly binding to the *oppC3* and *sdr3* promoters, and the citrate cycle by directly binding to the *sucA* promoter. In addition, MsmR1 regulated multiple biological processes, including carbohydrate metabolism, biofilm formation, chemotaxis, and motility. These results support the application of *Paenibacillus polymyxa* SC2 for biological control against various pathogenic bacteria in pepper.

In conclusion, transcriptional factors are essential for bacterial survival and adaptation. While many transcriptional factors have been identified, their functions are still poorly understood and may be diverse in different bacterial strains. Some new techniques still need to be developed to monitor the real-time transcriptional factor activity in living bacteria. Integrating data from multiple sources, such as transcriptomics, proteomics, and metabolomics, could provide a more comprehensive understanding of intricate regulation driven by transcriptional factors and their combinations. Better understanding of bacterial transcription factors can give insights into stresses adaptation, aid the development of new strategies to prevent bacterial infections, and promote the use of bacteria in biotechnology, agriculture, and other industries.

## Author contributions

DW and BL wrote the manuscript with input from all authors. All authors contributed to the article and approved the submitted version.

